# SmartBathroom Data Visualization Tool to Inform OT Clinical Reasoning

**DOI:** 10.1093/geroni/igab046.1644

**Published:** 2021-12-17

**Authors:** Jon Sanford, Avinandan Basu, Yangyi Xu

**Affiliations:** 1 Georgia Tech, Georgia Tech/Atlanta, Georgia, United States; 2 Georgia Tech, GA Tech/Atlanta, Georgia, United States

## Abstract

Traditionally, Occupational Therapy assessment of an older adult’s toilet transfer performance has been based on qualitative observation and client self-report. The purpose of this study was to evaluate the effectiveness of supplementing traditional clinical reasoning with quantitative transfer performance data about body and foot position, balance, hand placement and grasping forces on grab bars. Specifically, we conducted an online survey of occupational therapy practitioners and educators to assess the usefulness and usability of 2D and 3D graphic visualizations representing foot and hand position and forces exerted on the floor, toilet seat and grab bars. These data were captured by sensors located throughout GA Tech’s SmartBathroom laboratory during a study of transfer performance. Findings are being used to identify the most useful sensor data and the most effective ways to convey that data to improve training of occupational therapy students.

